# Covishield vaccine induces robust immune responses in Bangladeshi adults

**DOI:** 10.1016/j.ijregi.2022.04.006

**Published:** 2022-04-29

**Authors:** Taufiqur Rahman Bhuiyan, Marjahan Akhtar, Fatema Khaton, Sadia Isfat Ara Rahman, Jannatul Ferdous, A.S.M. Alamgir, Mahbubur Rahman, Zannat Kawser, Imrul Hasan, Stephen Beaven Calderwood, Jason B. Harris, Richelle C. Charles, Regina C. LaRocque, Edward Thomas Ryan, Sayera Banu, Tahmina Shirin, Firdausi Qadri

**Affiliations:** a International Centre for Diarrhoeal Disease Research Bangladesh, Dhaka, Bangladesh; b Institute of Epidemiology, Disease Control and Research, Dhaka, Bangladesh; c Institute of Developing Sciences and Health Initiatives, Dhaka, Bangladesh; d Division of Infectious Diseases, Massachusetts General Hospital, Boston, MA, USA; e Department of Medicine, Harvard Medical School, Boston, MA, USA; f Department of Pediatrics, Harvard Medical School, Boston, MA, USA; g Department of Immunology and Infectious Diseases, Harvard T.H. Chan School of Public Health, Boston, MA, USA

**Keywords:** Covishield, Seropositive, Antibodies, Bangladesh, Comorbidity

## Abstract

**Objective:**

To evaluate severe acute respiratory syndrome coronavirus-2 (SARS-CoV-2)-specific antibody responses after Covishield vaccination for 6 months after vaccination.

**Design:**

SARS-CoV-2-specific antibody responses were assessed by enzyme-linked immunosorbent assay of the recombinant receptor-binding domain of SARS-CoV-2 in 381 adults given the Covishield vaccine at baseline (*n*=119), 1 month (*n*=126) and 2 months (*n*=75) after the first dose, 1 month after the second dose (*n*=161), and monthly for 3 additional months.

**Results:**

Over 51% of participants were seropositive at baseline (before vaccination with Covishield), and almost all participants (159/161) became seropositive 1 month after the second dose. Antibody levels peaked 1 month after receipt of the second dose of vaccine, and decreased by 4 months after the first dose; the lowest responses were found 6 months after the first dose, although antibody responses and responder frequencies remained significantly higher compared with baseline (*P*<0.0001). Compared with younger participants, older participants had lower antibody responses 6 months after the first dose of vaccine (*P*<0.05). Participants who had previous SARS-CoV-2 infection showed robust higher antibody responses after vaccination.

**Conclusions:**

These findings help to elucidate the longevity of vaccine-specific antibody responses following vaccination with Covishield, and provide information relevant to the planning of booster doses after the initial two doses of vaccine.

## Highlights

• All participants became seropositive 2 months after receipt of the second dose of vaccine.
• Comparable antibody responses were observed in both males and females.
• Participants with previous severe acute respiratory syndrome coronavirus-2 infection showed a robust antibody response.
• Similar antibody responses were observed in participants with and without comorbidities.

## Introduction

The severe acute respiratory syndrome coronavirus-2 (SARS-CoV-2) pandemic had caused more than 300 million confirmed cases of coronavirus disease 2019 (COVID-19) and approximately 5.5 million deaths globally by the end of January 2022 (Worldometer, 2022). There is a desperate need for widely available and effective vaccines against COVID-19 (Dhama et al., 2020). Currently, there are at least 216 COVID-19 vaccine candidates in development, including 92 ongoing clinical trials (bioRENDER, 2022). Bangladesh has seven vaccines for COVID-19 under emergency use authorization (EUA).

In Bangladesh, the COVID-19 vaccination programme was initiated on 27 January 2021, and mass vaccination started on 7 February 2021 for all adults aged >40 years, including front-line healthcare workers aged >18 years; this programme was expanded to include school-age children (12–17 years) from November 2021 (Anonymous, 2021). For this programme, the Government of Bangladesh purchased 30 million vaccine doses (e.g. Covishield, Comirnaty, Spikevax, BBIBP-CorV) for the Bangladeshi population.

The use of adenovirus vector ChAdOx1 to deliver a COVID-19 vaccine (Covishield; Oxford/AstraZeneca COVID-19 AZD1222, Serum Institute of India) has EUA in Bangladesh, as well as the UK, the European Union, Argentina, Brazil, Dominican Republic, El Salvador, India, Malaysia, Mexico, Nepal, Pakistan, the Philippines, Sri Lanka and Taiwan. Studies have shown that the vaccine is safe and effective (Knoll and Wonodi, 2021; Voysey et al., 2021). A test-negative, case–control study conducted in India during the surge of the Delta variant showed that the effectiveness of two doses of Covishield vaccine against moderate-to-severe COVID-19 was 81.5% (95% confidence interval 9.9–99.0) (Thiruvengadam et al., 2022). Moreover, fully vaccinated people were highly protected against severe infection, hospitalization and death caused by the virus. Covishield vaccine appears to be immunogenic across all age groups >18 years. After vaccinaton, antibody responses against the SARS-CoV-2 spike protein were induced in adults (Ramasamy et al., 2021). Studies have shown that history of COVID-19 positivity impacts the magnitude and quality of the antibody response after COVID-19 vaccination; prior infection resulted in quicker and more robust vaccine-induced immune responses compared with participants who were infection-naïve (Tut et al., 2021). However, in most studies, immune responses were only measured at one or two time points after vaccination, so the impact of prior infection on overall kinetics and longevity of the responses is not known. As such, this study investigated the antibody response following Covishield vaccination in 381 Bangladeshi adults who were followed for 6 months. Immunoglobulin G (IgG) antibodies to the recombinant receptor binding domain (RBD) of SARS-CoV-2 were measured before vaccination, and the kinetics of antibody responses were followed longitudinally in participants, examining for differences in responses by age, gender, comorbidities and prior COVID-19 infection.

## Materials and methods

### Study design

This study was conducted at the International Centre for Diarrhoeal Disease Research, Bangladesh (icddr,b) and the Institute of Epidemiology, Disease Control and Research (IEDCR), Dhaka, Bangladesh. Participants had received the Covishield vaccine between February 2021 and April 2021 at government facilities in Bangladesh. One dose (0.5 mL) of the Covishield vaccine consists of 5 × 10^10^ viral particles of a recombinant, replication-deficient chimpanzee adenovirus vector encoding the SARS-CoV-2 spike glycoprotein, produced in genetically modified human embryonic kidney 293 cells (Serum Institute of India, 2021). Participants received two doses of the Covishield vaccine at an interval of 2 months, in accordance with guidelines from the Ministry of Health and Family Welfare of the Government of Bangladesh. Prior to enrolment, participants were interviewed and information regarding age, sex, prior COVID-19 infection and comorbidities was recorded. Based on history prior to vaccination, participants were grouped into laboratory-confirmed COVID-19-positive [COV-P; using real time polymerase chain reaction (RT-PCR)] and COVID-19-negative (COV-N) cohorts. Informed written consent was obtained from all participants. The Institutional Review Committee of icddr,b and IEDCR approved the study protocol.

### Specimen collection

Blood samples were collected from enrolled participants at seven different time points: day 0 (*n*=119, before the first dose of Covishield vaccine), day 30±10 (*n*=126, 1 month after the first dose), day 60±10 (*n*=75, 2 months after the first dose and before the second dose), day 90±10 (*n*=161, 3 months after the first dose and 1 month after the second dose), day 120±10 (*n*=32, 4 months after the first dose and 2 months after the second dose), day 150±10 (*n*=57, 5 months after the first dose and 3 months after the second dose) and day 180±10 (*n*=47, 6 months after the first dose and 4 months after the second dose). Serum was separated from blood after centrifugation at 700 x *g* for 15 min and then frozen (-80 °C) until laboratory analysis.

### SARS-CoV-2-specific enzyme-linked immunosorbent assay

The concentration of RBD-specific IgG antibody was determined using enzyme-linked immunosorbent assay (ELISA), as reported previously using an IgG-specific anti-RBD monoclonal antibody (Shirin et al., 2020; Akter et al., 2022; Bhuiyan et al., 2022a, Bhuiyan et al., 2022b). The RBD antigen of the spike protein of SARS-CoV-2 and the anti-RBD monoclonal antibody were gifts from A. Schmidt Lab, Ragon Institute, Boston, MA, USA (Iyer et al., 2020). Briefly, 96-well Nunc MaxiSorp plates (ThermoFisher, Waltham, MA, USA) were coated with 100 µL of SARS-CoV-2 RBD antigen (1 μg/mL in carbonate buffer) and incubated for 1 h at room temperature (RT). After 30 min of blocking with 5% non-fat milk at RT, heat-inactivated serially diluted serum samples were added to the plate, as described previously (Shirin et al., 2020; Akter et al., 2022), and incubated for 1 h at 37°C. Finally, peroxidase-conjugated secondary antibodies (goat anti-human IgG, Jackson ImmunoResearch, West Grove, PA, USA) were added to plates and incubated at ambient temperature for 30 min, followed by five washes with phosphate-buffered saline with Tween 20 and one wash with 1X phosphate buffered saline. Bound secondary antibodies were detected using ortho phenylenediamine (Sigma, St Louis, MO, USA) and 30% H_2_O_2_ after 20 min of incubation in the dark at RT. Optical density (OD) was measured at 450 nm and 570 nm in the Eon (Biotek) ELISA Reader; OD values were adjusted by subtracting 570 nm OD nm from the 450 nm OD. Based on pre-pandemic serum specimens collected from healthy individuals as well as sera obtained from patients with influenza, as discussed previously (Shirin et al., 2020; Akter et al., 2022), 500 ng/mL (0.5 µg/mL) was determined as the cut-off value for IgG seropositivity.

### Data analyses

This study analysed SARS-CoV-2 IgG-specific antibody responses in vaccinated participants in Bangladesh before and after Covishield vaccination. Statistical analysis was performed using the Mann-Whitney *U*-test. Graph Pad Prism Version 6.0 was used for generating plots and analyses.

## Results

### Study participants

In total, 381 healthy adults who received the Covishield vaccine in Dhaka, Bangladesh were enrolled in the study. One hundred and twenty-four (33%) participants were aged <40 years, all of whom were front-line workers who performed COVID-19-related work, and 257 (67%) participants were aged ≥40 years (Table 1). There were more male (64%, *n*=244) participants than female participants. Based on self-reported history prior to vaccination, 40% (*n*=152) of participants had had laboratory-confirmed COVID-19 (COV-P) in the 3–10 months before receiving the first dose of Covishield vaccine. Among the participants, 22% (*n*=84) did not provide their comorbidity history. Of the remaining participants, 38% (*n*=144) had a comorbid condition (e.g. asthma, diabetes, heart diseases, kidney disease, hypothyroidism and cancer; Table 1).

**Table 1:** Demographic information of the participants who received Covishield vaccine.

	*n* (%)
Participants enrolled	381
Sex	
Male	244 (64%)
Female	137 (36%)
Age (years)	
<40	124 (33%)
≥40	257 (67%)
COVID-19 history before vaccination	
Positive	152 (40%)
Negative	229 (60%)
Comorbidities^a^	
Unknown^b^	84 (22%)
No	153 (40%)
Yes	144 (38%)

COVID-19, coronavirus disease 2019.

a Including asthma, hypertension, diabetes, heart diseases, kidney disease, etc.

b Participants did not provide comorbidity data.

### SARS-CoV-2-specific IgG antibodies at baseline and after vaccination with Covishield vaccine

**Fig. 1:**
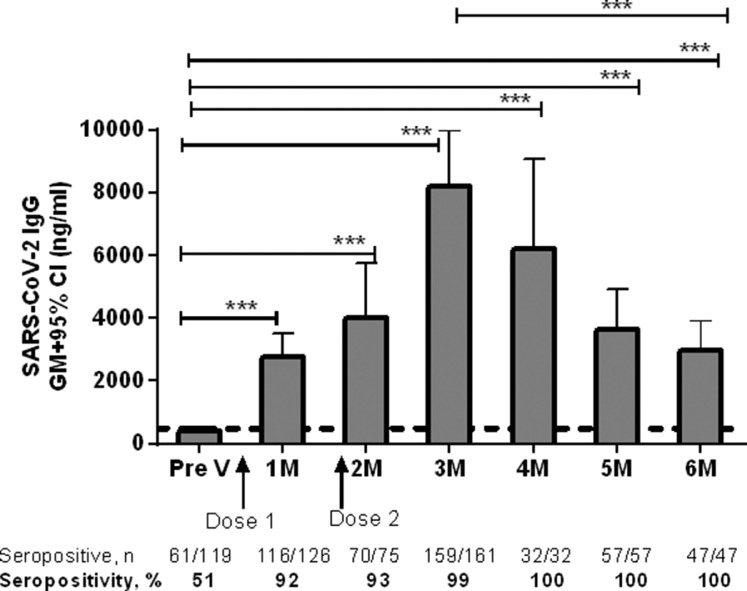
Level of severe acute respiratory syndrome coronavirus-2 (SARS-CoV-2) immunoglobulin (IgG) antibodies following Covishield vaccination in Bangladesh. Receptor-binding-domain-specific serum IgG antibody responses in participants receiving the Covishield vaccine. Levels of IgG antibodies were analysed using enzyme-linked immunosorbent assay before administration of the first dose of vaccine (Pre V, *n*=119), and 1 month (day 30±10, *n*=126), 2 months (day 60±10, *n*=75), 3 months (day 90±10, *n*=161), 4 months (day 120±10, *n*=32), 5 months (day 150±10, *n*=57) and 6 months (day 180±10, *n*=47) after the first dose. A second dose was given 2 months after the first dose. Bars indicate geometric mean (GM) concentrations of IgG with 95% confidence intervals (CI). The dashed line indicates the cut-off concentration of IgG antibody (500 ng/mL) for seropositivity. The number and percentage of seropositive individuals at each time point analysed are shown below the graph. *P<0.05, ^⁎⁎^*P*<0.01, ^⁎⁎⁎^*P*<0.001; ns, not significant.

Two doses of Covishield vaccine elicited strong antibody responses at all time points, and these decreased but remained significantly elevated up to 6 months following the first dose (Fig. 1). Before vaccination, 51% of the participants already had SARS-CoV-2 antibodies >500 ng/mL (cut-off concentration for seropositivity). Overall, at baseline, the geometric mean concentration of SARS-CoV-2 IgG was 403 ng/mL. One month after the first dose of vaccine, 92% of participants were seropositive and the responses were significantly higher (geometric mean 2727 ng/mL) compared with pre-vaccination levels (*P*<0.0001). The level of IgG antibodies increased even further (geometric mean 4029 ng/mL) 2 months after the first dose and before the second dose of vaccine (Fig. 1). One month after the second dose (3 months after the first dose), a strong response (geometric mean 8217 ng/mL) was seen, and almost 99% of the participants had RBD-IgG antibodies; at this time point, the highest magnitude of IgG antibody response was observed. Two months after the second dose (4 months after the first dose), 100% of the participants had seropositive levels of IgG antibodies above the 500 ng/mL cut-off, and these remained similar until 6 months after the first dose. Compared with baseline, IgG antibody responses remained significantly (*P*<0.0001) elevated up to 6 months after the first dose of vaccine; however, the antibody concentration had decreased (geometric mean 3271 ng/mL) by 6 months (*P*<0.0001) compared with the level observed 3 months after the first dose (Fig. 1).

### Effect of previous SARS-CoV-2 infection on Covishield-induced antibody responses

**Fig. 2:**
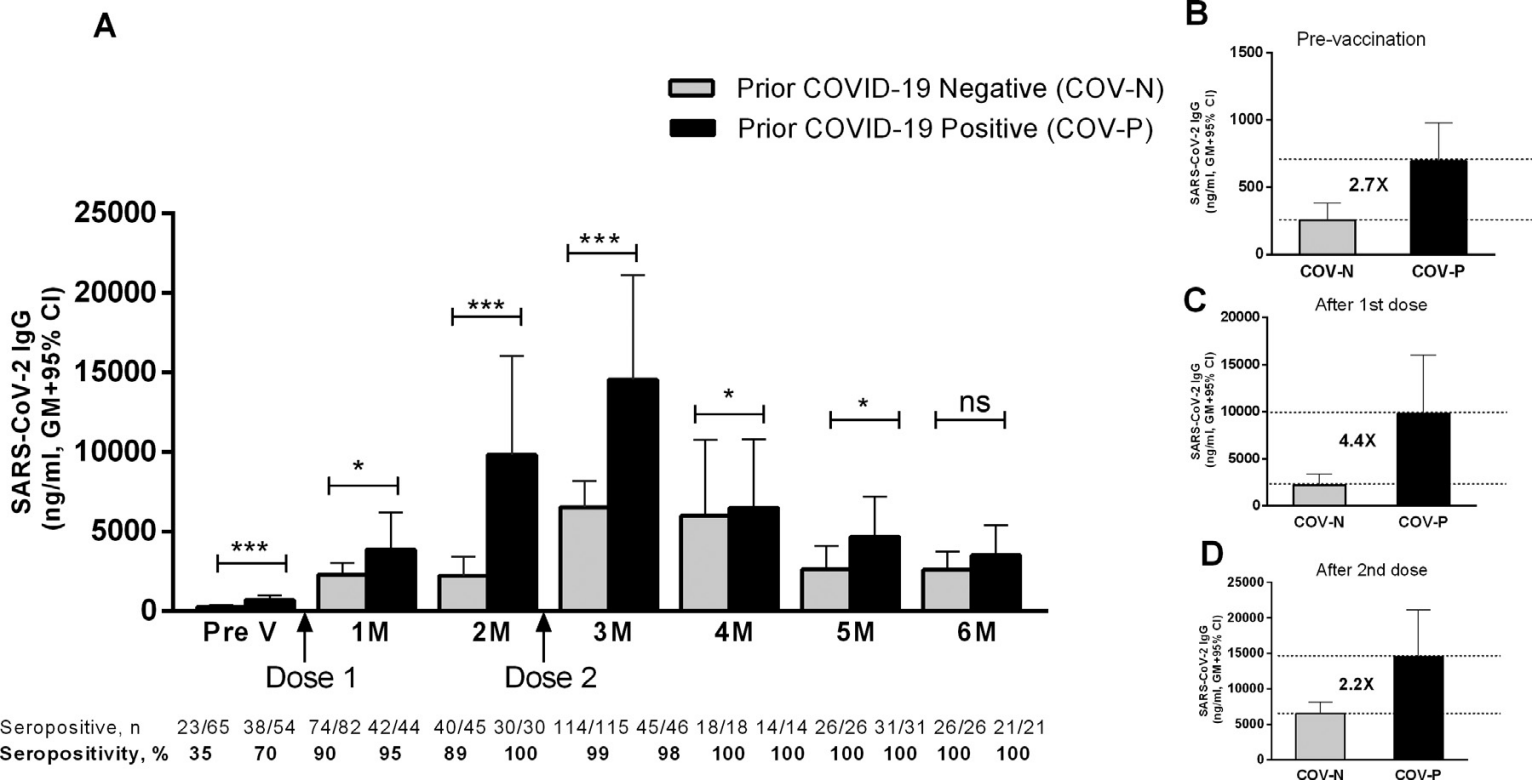
Immunoglobulin G (IgG) antibody responses after Covishield vaccination in prior coronavirus disease 2019 (COVID-19)-negative and -positive participants. (A) Receptor-binding-domain-specific serum IgG antibody response to Covishield vaccine in previously laboratory-confirmed COVID-19-positive participants (COV-P, black bar) and COVID-19-negative participants (COV-N, light bar). Levels of IgG antibodies were analysed using enzyme-linked immunosorbent assay before administration of the first dose of vaccine (Pre V, *n*=65 for COV-N, *n*=54 for COV-P) and again 1 month (*n*=82 for COV-N, *n*=44 for COV-P), 2 months (*n*=45 for COV-N, *n*=30 for COV-P), 3 months (*n*=115 for COV-N, *n*=46 for COV-P), 4 months (*n*=18 for COV-N, *n*=14 for COV-P), 5 months (*n*=26 for COV-N, *n*=31 for COV-P) and 6 months (*n*=26 for COV-N, *n*=21 for COV-P) after the first dose. A second dose was given 2 months after the first dose. Fold differences in the geometric mean antibody concentration between COV-N and COV-P at (B) pre-vaccination, (C) 2 months after the first dose of vaccine and before the second dose, and (D) 1 month after the second dose of vaccine (i.e. 3 months after the first dose) are shown using the dashed lines. Bars indicate the geometric mean (GM) concentration of IgG with 95% confidence interval (CI). The number and percentage of seropositive individuals in each time point analysed are shown below the graph. *P<0.05, ^⁎⁎^*P*<0.01, ^⁎⁎⁎^*P*<0.001. ns, not significant.

As many Bangladeshi participants had already been infected with SARS-CoV-2 before vaccination, antibody responses were compared between vaccinees who had no history of COVID-19 positivity (COV-N) and those who were previously COVID-19 positive (COV-P) by RT-PCR using nasopharyngeal swab specimens (Fig. 2A). The Covishield vaccine elicited strong IgG antibody responses in both groups. In the COV-N group, 35% of participants were seropositive for IgG at baseline (geometric mean 256 ng/mL). One month after the first dose of vaccine, 90% of the COV-N group became seropositive, and almost all became seropositive 2 months after receiving the second dose. Compared with baseline, significantly (*P*<0.0001) higher IgG antibody responses were found at all time points up to 6 months after the first dose of vaccine.

In contrast, 70% of the COV-P group were seropositive before the first dose of Covishield vaccine, but their antibody titres increased significantly after receiving the second dose (Fig. 2A). At baseline, the IgG level was significantly higher (*P*<0.001, 2.7-fold higher geometric mean, Fig. 2B) in the COV-P group compared with the COV-N group. After receiving the first and second doses of the Covishield vaccine, IgG concentrations were significantly higher (*P*<0.05 to *P*<0.0001) in the COV-P group at all time points up to 3 months after the first dose (one month after the second dose). Before giving the second dose (i.e. 2 months after the first dose), the COV-P group had antibody responses higher than the COV-N group at any timepoint (Fig. 2C). However, in both groups, the highest vaccine-induced antibody responses occurred 1 month after the second dose (i.e. 3 months after the first dose) (Fig. 2A), although the COV-P group responded with 2.2-fold higher levels compared with the COV-N group at that time point (Fig. 2D). One month later, IgG levels decreased significantly (*P*<0.05) in the COV-P group, but were still higher (*P*<0.05) compared with the COV-N group. Six months after the first dose of vaccine, antibody levels decreased further in both the COV-P and COV-N groups, and no differences were observed between them at that time point. However, the fold difference in the geometric mean IgG concentration (post-vaccination/pre-vaccination) was higher in the COV-N group compared with the COV-P group.

Compared with pre-vaccination levels, the geometric mean increase in antibody levels 4 months after receipt of the first dose of Covishield vaccine was 9.3 fold for the COV-P group and 23.5-fold for the COV-N group.

### Vaccine-induced antibody responses based on gender and age

**Fig. 3:**
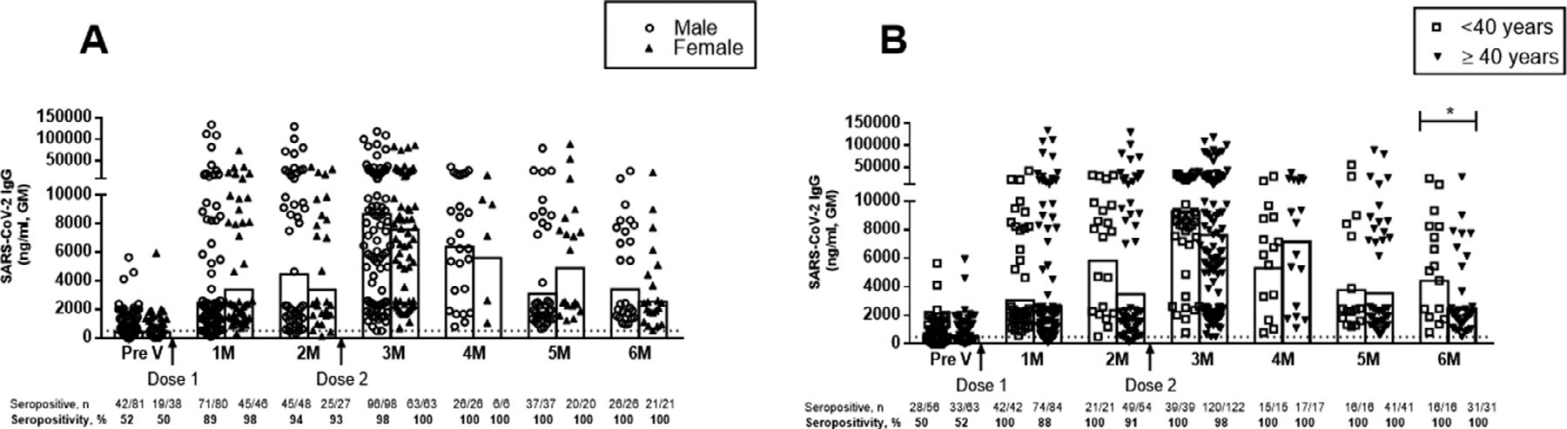
Covishield-induced antibody responses in different gender and age groups. Receptor-binding-domain-specific serum immunoglobulin G (IgG) antibody responses in (A) male and female participants before the first dose of vaccine (Pre V, *n*=81 male, *n*=38 female), and 1 month (n=80 male, *n*=46 female), 2 months (*n*=48 male, *n*=27 female), 3 months (*n*=98 male and *n*=63 female), 4 months (*n*=26 male, *n*=6 female), 5 months (*n*=37 male, *n*=20 female) and 6 months (*n*=26 male, *n*=21 female) after the first dose. (B) Vaccine-induced IgG antibodies in participants aged <40 years vs ≥40 years before the first dose (*n*=56 vs 63), and 1 month (*n*=42 vs 84), 2 months (*n*=21 vs 54), 3 months (*n*=39 vs 122), 4 months (*n*=15 vs 17), 5 months (*n*=16 vs 41) and 6 months (*n*=16 vs 31) after the first dose. A second dose was given 2 months after the first dose. Each symbol indicates data from one participant, and bars represent geometric mean (GM) concentrations of IgG. The dashed line indicates the cut-off concentration of IgG antibody (500 ng/mL) for seropositivity. Number and percentage of seropositive individuals in each time points analysed are shown below the graph. *P<0.05, ^⁎⁎^*P*<0.01, ^⁎⁎⁎^*P*<0.001. ns, not significant.

The IgG antibody responses were analysed between males and females after Covishield vaccination. No significant difference was observed (*P*>0.05) between males and females at any time points after vaccination (Fig. 3A).

Next, vaccine induced IgG antibodies were analysed in participants in two age groups: <40 years and ≥40 years (Fig. 3B). A comparable increase in SARS-CoV-2-specific IgG antibodies was observed in both age groups. No significant differences were observed (*P*>0.05) between the age groups until 5 months after the first dose of Covishield vaccine. By 6 months after the first dose, participants aged <40 years had a significantly higher level of antibody response (*P*<0.05) compared with participants aged ≥40 years (Fig. 3B). However, both age groups were still 100% seropositive at 6 months after initial vaccination. Furthermore, this difference was based on a relatively small sample size compared with earlier timepoints.

**Fig. 4:**
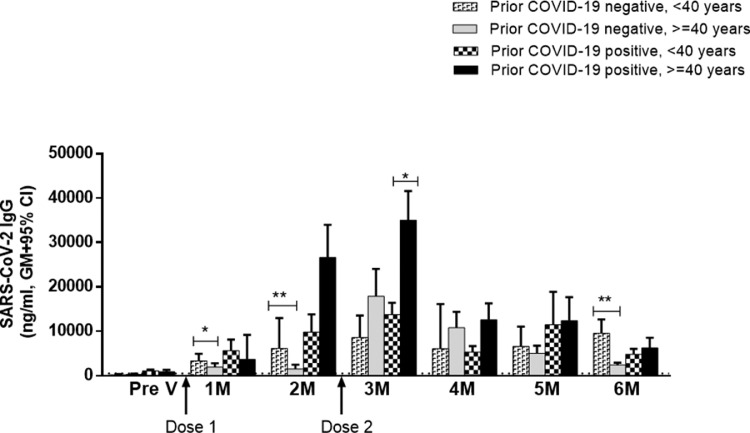
Covishield-induced severe acute respiratory syndrome coronavirus-2 (SARS-CoV-2) immunoglobulin (IgG) antibodies in prior coronavirus disease 2019 (COVID-19)-positive and -negative participants from older and younger age groups. Receptor-binding-domain-specific serum IgG antibody responses were measured in four different groups: (1) <40 years and prior COVID negative (*n*=6–29), (2) ≥40 years and prior COVID negative (*n*=10–93), (3) <40 years and prior COVID positive (*n*=6–27) and (4) ≥40 years and prior COVID positive (*n*=7–28) before vaccination and every month until 6 months after the first dose of vaccine. A second dose was given 2 months after the first dose. Bars indicate the geometric mean (GM) concentration of IgG with 95% confidence interval (CI). The dashed line indicates the cut-off concentration of IgG antibodies (500 ng/mL) for seropositivity. *P<0.05, ^⁎⁎^*P*<0.01.

This study also compared age-specific vaccine responses in the COV-P and COV-N groups (Fig. 4). In the COV-N group, participants aged <40 years had significantly higher IgG antibodies compared with those aged ≥40 years 1 month (*P*<0.05) and 2 months (*P*<0.001) after the first dose of vaccine. However, after the second dose, responses were comparable in both age groups within 3–5 months of vaccination. Again, 6 months after the first dose of vaccine, COV-N participants aged <40 years had higher antibody levels compared with those aged ≥40 years, which confirms the importance of people aged ≥40 years receiving a booster dose 6 months after the first dose of vaccine.

In contrast, among the COV-P participants, vaccine responses were comparable in both age groups after the first dose of Covishield vaccine. However, 1 month after the second dose, significantly higher (*P*<0.05) antibody responses were observed in those aged ≥40 years compared with those aged <40 years (Fig. 4).

### Impact of comorbidity on levels of SARS-CoV-2 IgG antibodies

The authors were interested to determine if there were any differences in Covishield-induced antibody responses between participants who had comorbid diseases compared with those who did not. In comparison with the pre-vaccination (baseline) level, regardless of comorbidity status, Covishield induced a significant (*P*<0.0001) increase in IgG antibodies in both groups at all time points after vaccination. Participants with comorbidities had slightly higher baseline IgG seropositivity compared with participants without comorbidities (58% vs 49%, data not shown), although the rate of COVID positivity prior to vaccination was comparable in participants with (53%) and without (57%) comorbidities. However, no significant differences in SARS-CoV-2 IgG levels were observed between participants with comorbidities and those without comorbidities at any subsequent time points (*P*>0.05).

## Discussion

This study found that two doses of the Covishield vaccine given at an 8-week interval generates significant SARS-CoV-2-specific antibody responses in adults in Bangladesh. Participants who reported recent SARS-CoV-2 infection had higher levels of SARS-CoV-2 RBD antibodies following vaccination than those without a history of infection. However, the gap between the COV-P and COV-N groups was highest after the first dose of vaccine. Ultimately, 6 months after the first dose of vaccine, after receiving a second dose of the vaccine, there was no significant difference in titres between those with and without evidence of prior SARS-CoV-2 infection.

Without an established immune correlate of protection for SARS-CoV-2 vaccines, as well as the continued emergence of new SARS-CoV-2 variants, the protective efficacy of vaccination cannot be inferred precisely from these results. However, the antibody findings are consistent with other studies which suggested that repeated exposures to both vaccination and natural infection (e.g. hybrid immunity) serve to increase protection associated with vaccination (Hammerman et al., 2022). However, the present study suggests that the immunologic benefits of hybrid immunity may be mitigated over time and by repeated doses of vaccine. In other words, the immunologic benefits of mixed exposure to vaccination and natural infection may be reduced over time if several booster doses of vaccine are given.

Recently, a British drug manufacturer reported that a third booster shot of the Vaxzevria vaccine (which is the same as Covishield) significantly boosts antibody levels against the Omicron variant of SARS-CoV-2. Some experts are also of the opinion that mixing and matching vaccine shots may give better results to boost immunity (Shaw et al., 2021; Costa Clemens et al., 2022; Nguyen et al., 2022). However, AstraZeneca has advocated the use of Vaxzevria as a third booster dose against Omicron (Anonymous, 2022). If the vaccine is recommended for use as a booster against Omicron, this may significantly increase the demand for Covishield in India.

Different comorbid conditions (e.g. hypertension, cardiac disease, kidney disease, diabetes mellitus etc.) were also analyed for observing associations with differing responses following COVID-19 vaccination. However, comorbidities did not appear to have any negative effect on mounting immune responses after Covishield vaccination. In addition, there was no significant difference in antibody responses after the second dose between participants who had a comorbid condition and those who did not. However, another study reported lower antibody levels among comorbid individuals compared with those without comorbidities after the second dose of Vaxzevria, although the difference in antibody levels between participants with comorbidities [median 10.60, interquartile range (IQR) 8.21–11.90] and without comorbidities (median 10.60, IQR 8.21–11.90) was very small (Hoque et al., 2021).

The results of this study re-emphasize the urgent need to deploy the most effective vaccine strategies as widely and rapidly as possible in order to protect the population against the different emerging lineages of concern of SARS-CoV-2. The Government of Bangladesh is currently trying to cover the population by mass vaccination campaigns in different divisions, cities and wards over the country. Policy makers are continuously observing outcomes from different countries, and implementing improvements in the vaccine administration strategies in Bangladesh. It is hoped that the present findings will help policy makers to understand the immune response to the Covishield vaccine over time and in different age groups, by gender and by comorbidities. Several ongoing studies are investigating the effects of mixing different COVID-19 vaccine formulations, including for booster doses. Data have been released from mixed trials in Asian and European countries which suggest that mixing vaccines leads to a stronger immune response, and sometimes outperforms two doses of the same vaccine (Callaway, 2021; Maccioni, 2021; Melimopoulos, 2021; Shaw et al., 2021). This study also observed waning of antibody responses in a few participants after Covishield vaccination, and a small percentage of participants acquired infection. More in-depth analyses (e.g. neutralizing antibody responses, T-cell responses and variant analyses) are needed in future studies to understand the vaccine escape phenomena. Not all vaccines are equally protective against all variants (Madhi et al., 2021), and this is important in understanding vaccine escape mechanisms.

## Conflict of interest statement

None declared.
